# Scorpion in Combination with Gypsum: Novel Antidiabetic Activities in Streptozotocin-Induced Diabetic Mice by Up-Regulating Pancreatic PPAR**γ** and PDX-1 Expressions

**DOI:** 10.1093/ecam/neq031

**Published:** 2011-04-14

**Authors:** Weidong Xie, Yunan Zhao, Dayong Gu, Lijun Du, Guoping Cai, Yaou Zhang

**Affiliations:** ^1^Life Science Division, Graduate School at Shenzhen, Tsinghua University, Shenzhen 518055, China; ^2^Laboratory of Pathological Sciences, Basic Medical College, Nanjing University of Traditional Chinese Medicine, Nanjing 210029, China; ^3^Institute of Disease Control and Prevention, Shenzhen International Travel Health Care Center, Shenzhen Entry-Exit Inspection and Quarantine Bureau, Shenzhen 518045, China; ^4^Laboratory of Pharmaceutical Sciences, Department of Biological Sciences and Biotechnology, Tsinghua University, Beijing 100084, China

## Abstract

The management of diabetes without any side effects remains a challenge in medicine. In this study, antidiabetic activity and the mechanism of action of scorpion combined with gypsum (SG) were investigated. Streptozotocin-induced diabetic mice were orally administrated with scorpion (200 mg kg^−1^ per day) in combination with gypsum (200 mg kg^−1^ per day) for 5 weeks. SG treatment resulted in decreased body weight, blood glucose and lipid levels, and increased serum and pancreatic insulin levels in diabetic mice. Furthermore, SG significantly increased the number and volume of beta cells in the Islets of Langerhans and promoted peroxisome proliferator-activated receptor gamma and pancreatic duodenal homeobox 1 expressions in pancreatic tissues. However, scorpion or gypsum alone had no significant effect in this animal model. Metformin showed a slight or moderate effect in this diabetic model, but this effect was weak compared with that of SG. Taken together, SG showed a new antidiabetic effect in streptozotocin-induced diabetic mice. This effect may possibly be involved in enhancing beta-cell regeneration and promoting insulin secretion by targeting PPAR**γ** and PDX-1. Moreover, this new effect of SG offers a promising step toward the treatment of diabetic patients with beta-cell failure as a complementary and alternative medicine.

## 1. Introduction

In the coming years, the number of people with diabetes in the world will more than double, from 171 million in 2000 to 366 million in 2030 [1]. In 2007, >110 million individuals in Asia were living with diabetes [2]. Diabetes mellitus is associated with the development of micro- and macrovascular complications, which are the major causes of morbidity and death. Treatment of diabetes mellitus usually involves diet control, exercise and the use of hypoglycemic diets and drugs. However, many oral medicines have a number of adverse effects [3]. The management of diabetes without any side effects continues to remain a challenge to the medical system. Type 1 diabetic and terminal type 2 diabetic patients still depend largely on insulin treatment. However, insulin has limitations in terms of treatment, such as hypoglycemia, allergic reaction, insulin intolerance and inconveniences related to injection. Beta-islet dysfunction might be a key to the development of diabetes mellitus [4]. No special antidiabetic drugs have yet to show that the activities of promoting *β*-islet regeneration, though peroxisome proliferator-activated receptor gamma (PPAR*γ*) agonists and incretin-mimetic agents have the most promising action in the protection of beta cells [5].

The application of Chinese medicines for antidiabetic activities have been recently reviewed [6–9]. Most of these drugs are derived from herb extracts or herbal formulas, and are widely used by patients with diabetes mellitus. Most have been found to have a positive effect on hyperglycemia and diabetic complications. The use of Chinese herbal medicines in diabetes is promising, although its effectiveness has yet to be fully proven [10]. However, there are clinical limitations to the application of Chinese medicines in diabetic patients because of inappropriate use (or combination use) or the lack of scientific and systematic evidence. In addition, most therapeutic plans still focus on lowering blood glucose levels or the number of diabetic complications. Indeed, protection or regeneration of beta cells should be considered the key strategies to the treatment of diabetes mellitus.

Scorpion or gypsum in combination with other Chinese medicines is mainly used to treat diabetic neuropathy [11] or ameliorate the syndrome of thirst [12]. At present, however, there have been no systematic and scientific trials conducted to study their antidiabetic activities. To the authors' knowledge, neither scorpion nor gypsum alone has significant hypoglycemic effects. In this study, a combination of two Chinese medicines, scorpion and gypsum (SG), appeared to have potential antidiabetic properties mediated by promoting the regeneration of beta islet cells.

## 2. Methods

### 2.1. Preparation of Extracts

Scorpion (*Buthus martensii kirsch*, Quanxie, Batch No. 20090314) and gypsum (Shigao, Batch No. 20090118) were obtained from the Shenzhen Leepheng Medicine Co., Ltd. To lower the toxic effect, we discarded the toxic sting in the tail of the scorpion before its whole body was used for further preparations. First, the scorpion was dried overnight at 60°C and then reduced to a powder. Gypsum was added to distilled water (1 g : 5 ml) and cooked at *∼*95°C for 1 h. Aqueous extracts of gypsum were filtered through a cotton gauze. The residual precipitates of gypsum were discarded (*∼*90%). The powdered scorpion was combined with the aqueous extract of gypsum (200 mg scorpion and 200 mg gypsum in 1 ml), slowly cooled at room temperature and stored at 4°C until further use. Extracts of scorpion or gypsum alone were also prepared. Gypsum solution was substituted with water for the scorpion extract; no scorpion powder was added to the gypsum solution.

### 2.2. Animals and Diets

Male NIH mice (SPF grade, Certified No. SCXK(Guangdong) 2003-0002) were obtained from the Guangdong Medical Laboratory Animal Center (Guangzhou, Guangdong, China). Animals were kept in an environmentally controlled breeding room (temperature: 20 ± 2°C, humidity: 60 ± 5%, 12-h dark/light cycle). They were fed standard laboratory chow with water *ad libitum* and were fasted from 9:00 a.m. to 3:00 p.m. before the experiments. The research was conducted in accordance with the Declaration of Helsinki and the Guide for the Care and Use of Laboratory Animals as adopted and promulgated by the United States National Institutes of Health and approved by the Animal ethics Committee of Tsinghua University, China. Normal chow diets were obtained from the Guangdong Medical Laboratory Animal Center. These contained crude protein (20%), crude fat (4%) and crude carbohydrates (60%) (g g^−1^).

### 2.3. Preparation of Diabetic Mouse Model

Male NIH mice weighing 18–20 g were selected for this study. First, the animals were subjected to fasting for 24 h but were given free access to water. Diabetes was subsequently induced in the animals through intraperitoneal (ip) administration of streptozotocin (STZ, Sigma, USA) at a dose of 100 mg kg^−1^ body weight. STZ was freshly prepared in an ice-cold citrate buffer (0.1 mmol l^−1^, pH 4.5) and immediately injected into the animals (within 5 min). A week later, high and steady blood glucose levels were observed in STZ-induced mice. At this point, the STZ-induced mice with high blood glucose levels (>12 mmol l^−1^) were selected as diabetic models.

### 2.4. Experimental Procedure

Normal mice were randomly divided into two subgroups (*n* = 10): normal control mice and normal mice treated with the scorpion extracts (350 mg kg^−1^ per day) combined with gypsum (350 mg kg^−1^ per day). STZ-treated diabetic mice were randomly divided into five subgroups (*n* = 10): Group 1, diabetic control mice; Group 2, diabetic mice treated with scorpion (350 mg kg^−1^ per day) combined with gypsum (350 mg kg^−1^ per day); Group 3, diabetic mice treated with scorpion (350 mg kg^−1^ per day); Group 4, diabetic mice treated with gypsum (350 mg kg^−1^ per day) and Group 5, diabetic mice treated with metformin (250 mg kg^−1^ per day). The tested samples were administered orally. Normal and diabetic controls received distilled water with similar volume (0.1 ml/10 g body weight). The administration of extracts was continued for 5 weeks, once daily. In a separate trial, after 5 weeks of treatment, the treatment was terminated for 1 week to determine whether the blood glucose level increased in mice. Blood samples were collected from tail veins at 6 h after the administration of extracts for biochemical assaying. The anesthetized mice (ip injection of pentobarbital at a dose of 35 mg kg^−1^) were sacrificed by cervical dislocation after their blood was collected. Serum was separated for biochemical assays. Pancreatic tissue was removed and frozen immediately in liquid nitrogen for biochemical analysis. Fresh pancreatic tissue was fixed in 10% formalin solution for 48 h for histopathological examination.

### 2.5. Histopathology

Pancreatic tissue was processed for routine paraffin-wax histology and sections were stained with hematoxylin and eosin (H&E). Five non-overlapping adjacent sections were observed for the Islets of Langerhans to measure the diameter from each group sample.

### 2.6. Oral Glucose Tolerance Test

Oral glucose-tolerance test was conducted as prescribed previously with a slight modification [13]. In a separate trial, four groups of animals were adopted (*n* = 6): normal, diabetic control mice, diabetic mice treated with scorpion (350 mg kg^−1^ per day) combined with gypsum (350 mg kg^−1^ per day) and diabetic mice treated with metformin (250 mg kg^−1^ per day). The mice were fasted for 6 h following 5 weeks of treatment. Using a light anesthesia through an ip injection of pentobarbital at a dose of 35 mg kg^−1^, blood samples were collected from the tail vein of the mice for assaying blood glucose levels at 0, 0.5, 1 and 2 h after glucose (2.5 g kg^−1^) administration. Blood glucose levels were determined using a commercial kit (BioSino Bio-technology and Science Inc, Beijing, China). The curve of blood glucose concentration and time was plotted. Area under circle (AUC) of blood glucose and time were calculated according to the formula: AUC_0−2 h_ = [(*G*_0 h_ + *G*_0.5 h_) × 0.5 h + (*G*_0.5 h_ + *G*_1 h_) × 0.5 h + (*G*_1 h_ + *G*_2 h_) × 1 h]/2; “*G*”, blood glucose value.

### 2.7. Intraperitoneal Insulin-Tolerance Test

Insulin sensitivity was assayed through ip insulin-tolerance test according to the previous method, but with a slight modification [14]. Four groups of animals were adopted in a separate trial (*n* = 6): normal, diabetic control mice, diabetic mice treated with scorpion (350 mg kg^−1^ per day) combined with gypsum (350 mg kg^−1^ per day) and diabetic mice treated with metformin (250 mg kg^−1^ per day). After 5 weeks of treatment, the mice were fasted for 6 h and anesthetized with an ip injection of pentobarbital at a dose of 35 mg kg^−1^, and then received an ip injection of short-effect human insulin (Novo Nordisk, North Carolina, USA) at a dose of 0.2 U kg^−1^. Blood was collected from the tail vein for blood glucose assaying after 0, 0.5 and 1 h of insulin treatment. The decrease in the percentage of blood glucose concentration (versus baseline (0 h)) was calculated. All values were multiplied by (−1) and a statistical analysis was conducted.

### 2.8. Biochemical Analysis

Blood glucose and triglycerides, total cholesterol, low-density lipoprotein cholesterol (LDL-c) and high-density lipoprotein cholesterol (HDL-c) (*n* = 10) levels were estimated using commercial kits [BioSino Bio-technology and Science Inc, Beijing, China [15–19]]. Serum and pancreatic insulin levels were determined using the ABC-ELISA kit (Shanghai Westang Bio-tech, China).

### 2.9. Western Blotting

Freshly prepared whole pancreatic tissues (*n* = 5) were homogenated and lysed with NETN buffer (20 mM Tris-HCl, pH 7.8, 1 mM EDTA, 50 mM sodium chloride and 0.5% NP-40). Lysates were centrifuged at 12 000 rpm at 4°C for 2–10 min. Supernatants were collected, and protein concentration was determined using the BCA assay kit (Nanjing Jiancheng Biotech, China). Western blotting analysis was carried out according to the manufacturer's protocol. Antibodies against PPAR*γ* (Wuhan Boster Bio-Tech, China), pancreatic duodenal homeobox 1 (PDX-1, Shanghai Ruicong Bio-tech, China) and glyceraldehyde-3-phosphate dehydrogenase (GAPDH) (Biolegend; 1 : 500–1000) were used. Protein expression was visualized with horseradish peroxidase-conjugated secondary antibodies (Amersham Biosciences, USA; 1 : 2000) and enhanced chemiluminescence (KPL, USA).

### 2.10. Data Analysis

Data were expressed as mean ± standard deviation (SD). Statistical analysis was performed using one-way analysis of variance (ANOVA). The Newman-Keuls comparisons were used to determine the source of significant differences where appropriate. *P*-values < .05 were considered statistically significant.

## 3. Results

### 3.1. Blood Glucose Levels

**Figure 1:**
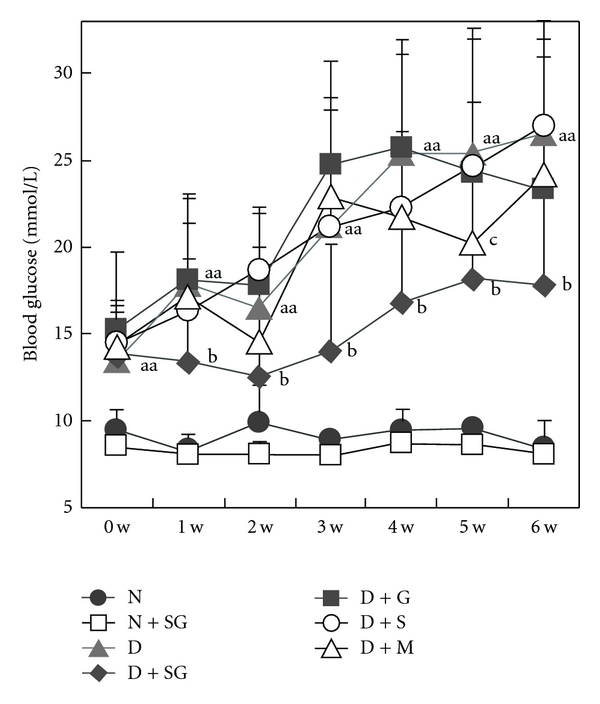
Changes in fasting blood glucose levels with time (0–6 weeks) in mice. To be specific, the tested samples were continually given for 5 weeks and then cancelled for 1 week. N, Normal control mice; N + SG, normal mice treated with the aqueous extracts of scorpion (350 mg kg^−1^ per day) combined with gypsum (350 mg kg^−1^ per day); D, diabetic control mice; D + SG, diabetic mice treated with scorpion (350 mg kg^−1^ per day) combined with gypsum (350 mg kg^−1^ per day); D + G, diabetic mice treated with gypsum (350 mg kg^−1^ per day); D + S, diabetic mice treated with scorpion (350 mg kg^−1^ per day); D + M, diabetic mice treated with metformin (250 mg kg^−1^ per day). Data were expressed as mean ± SD (*n* = 10). ^aa^ *P* < .01 “D” versus “N”; ^b^ *P* < .05 “D + SG” versus “D”; ^c^ *P* < .05 “D + M” versus “D”.

STZ-induced diabetic mice showed a significant increase in blood glucose levels compared with normal controls during the period (Figure 1). However, this increase was significantly inhibited by SG during the 5-week period of administration. Neither scorpion nor gypsum alone had significant antidiabetic effects, which suggested that SG might have a synergic anti-hyperglycemic effect. However, SG had no hypoglycemic effect in normal mice. Metformin showed a significant hypoglycemic effect only at the fifth week, which suggested that metformin might be inappropriate for the treatment of severe hyperglycemia, or that its effects might surface in a slow manner. Furthermore, the treatment was stopped for 1 week after 5 weeks of treatment to determine if blood glucose levels would increase in diabetic mice. Interestingly, SG still had a hypoglycemic effect in diabetic mice. However, blood glucose levels in metformin-treated mice showed a rapid rebound. Therefore, we hypothesized that SG had a fundamental effect in this diabetic model, which was associated with a protective effect on beta-islet cells and resulted in the normalization of blood glucose level.

### 3.2. Serum Lipid Levels

Diabetic mice showed a significant increase in serum triglyceride and decrease in HDL-C compared with normal controls after 5 weeks of treatment (Table 1). Serum total cholesterol and LDL-C showed an increasing trend but had no significant changes as compared with those in normal controls. Despite this, total cholesterol/HDL-C ratios were significantly increased in diabetic mice compared with those in normal controls. After 5 weeks of treatment, SG normalized diabetic dyslipidemia in STZ-induced mice to the full extent but had no effect in normal mice. This effect might be a secondary response to antidiabetic effects. Metformin appeared to have no significant effect on lipid regulation in this diabetic model.

### 3.3. Body Weight, Water and Diet Intake

Diabetic mice had significantly decreased body weight despite an increase in both water and diet intakes (Table 2). These symptoms are similar to type 1 diabetic patients. SG significantly inhibited the decrease of body weight and resisted the increase in both water and diet intakes in diabetic mice. Scorpion alone effected a significant increase in the body weights of diabetic mice but had no significant effect on both water and diet intakes. Gypsum alone, on the other hand, effected a significant decrease in water intake but had no significant effect on body-weight gain and diet intake. Metformin effected a significant increase in body weight and a decrease of water intake but had no effect on diet intake. These results further supported the supposition that SG had significant antidiabetic effects.

### 3.4. Serum and Pancreatic Tissue Insulin Levels

**Figure 2:**
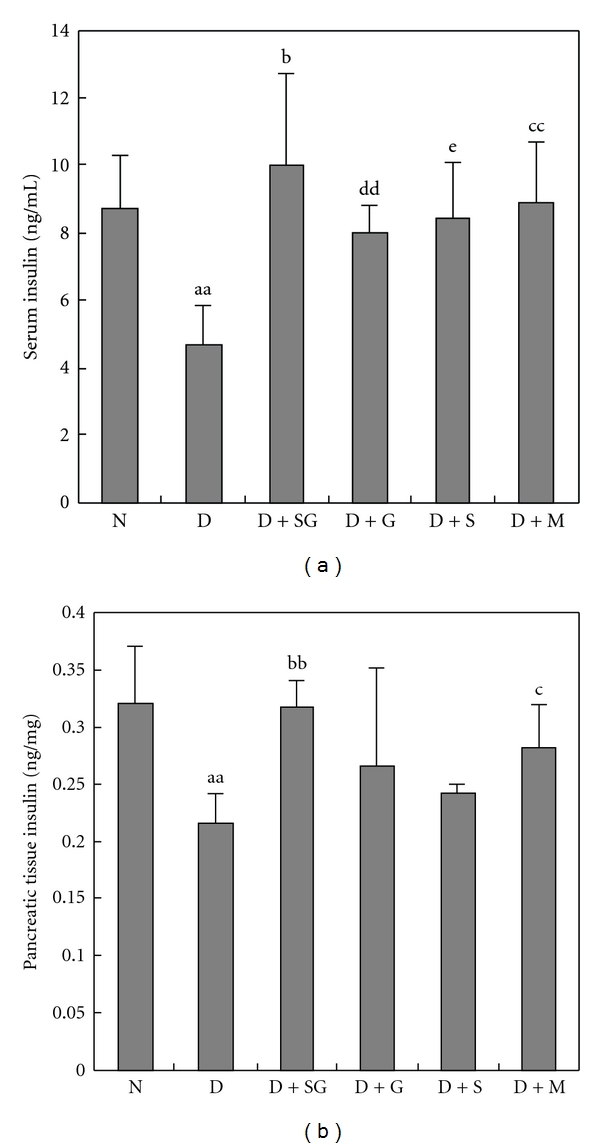
Serum and pancreatic tissue insulin levels detected by ELISA after 5 weeks of treatment (a and b). N, Normal control mice; D, diabetic control mice; D + SG, diabetic mice treated with scorpion (350 mg kg^−1^ per day) combined with gypsum (350 mg kg^−1^ per day); D + G, diabetic mice treated with gypsum (350 mg kg^−1^ per day); D + S, diabetic mice treated with scorpion (350 mg kg^−1^ per day); D + M, diabetic mice treated with metformin (250 mg kg^−1^ per day). Data were expressed as mean ± SD (*n* = 5). ^aa^ *P* < .01 “D” versus “N”; ^b^ *P* < .05,  ^bb^ *P* < .01 “D + SG” versus “D”; ^dd^ *P* < .01 “D + G” versus “D”; ^e^ *P* < .05 “D + S” versus “D”.

There was a significant decrease of serum and pancreatic tissue insulin levels in diabetic mice compared with those in normal controls (Figure 2). This decrease was significantly inhibited in diabetic mice treated with SG or metformin, respectively. Moreover, scorpion or gypsum alone had the effect of a moderate increase in serum insulin levels but had no significant effect on pancreatic insulin levels. These results indicated that SG might promote insulin synthesis and release. Metformin appeared to possess the same potential but was weaker than SG.

### 3.5. Histopathology

**Figure 3:**
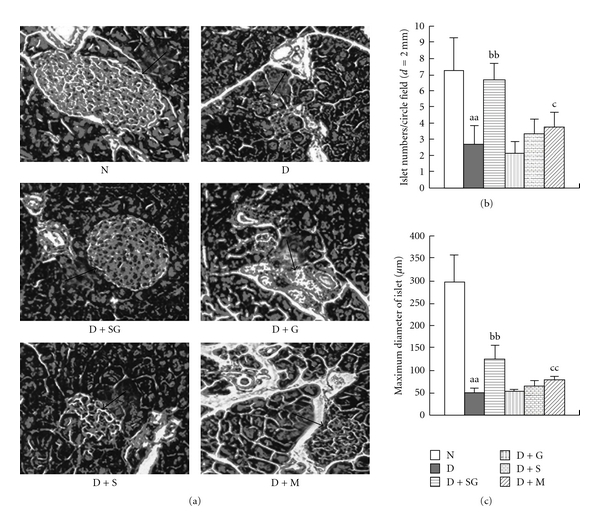
Images of H&E staining (×100 magnification) in pancreatic tissues of mice (a), pancreatic islet numbers (b), and maximum diameter of the Islets of Langerhans (c) (*n* = 5). Arrows indicate the Islets of Langerhans. N, Normal control mice; D, diabetic control mice; D + SG, diabetic mice treated with scorpion (350 mg kg^−1^ per day) combined with gypsum (350 mg kg^−1^ per day); D + G, diabetic mice treated with gypsum (350 mg kg^−1^ per day); D + S, diabetic mice treated with scorpion (350 mg kg^−1^ per day); D + M, diabetic mice treated with metformin (250 mg kg^−1^ per day). Data were expressed as mean ± SD (*n* = 5). ^aa^ *P* < .01 “D” versus “N”; ^bb^ *P* < .01 “D + SG” versus “D”; ^c^ *P* < .05, ^cc^ *P* < .01 “D + M” versus “D”.

Histopathological images of H&E-stained pancreatic tissues showed that the number and volume of Islets of Langerhans cells were significantly reduced in diabetic mice (Figure 3). The reduction was significantly inhibited in diabetic mice after SG administration. Scorpion or gypsum alone had no significant effect on the regeneration of pancreatic beta cells. Metformin also had a slight protective effect on pancreatic beta cells. However, SG displayed a higher increase in both number and volume of beta cells in the Islets of Langerhans in diabetic mice than metformin. These results suggested that SG might exert a hypoglycemic effect by promoting the regeneration of pancreatic beta cells.

### 3.6. Oral Glucose Tolerance Test

**Figure 4:**
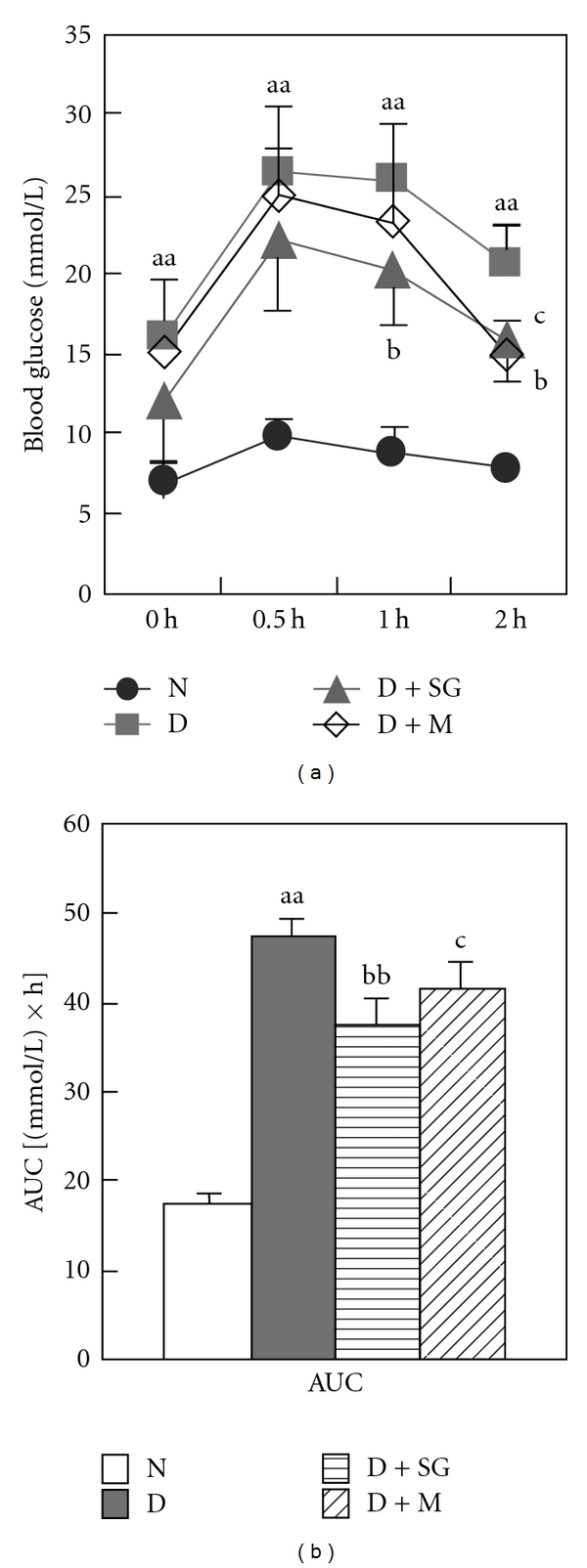
Changes in blood glucose levels with time (a) and areas under the circle (AUC) of blood glucose and time (b) after oral glucose load (2.5 g kg^−1^). N, Normal control mice; D, diabetic control mice; D + SG, diabetic mice treated with scorpion (350 mg kg^−1^ per day) combined with gypsum (350 mg kg^−1^ per day); D + M, diabetic mice treated with metformin (250 mg kg^−1^ per day). Data were expressed as mean ± SD (*n* = 6). ^aa^ *P* < .01 “D” versus “N”; ^b^ *P* < .05, ^bb^ *P* < .01 “D + SG” versus “D”; ^c^ *P* < .05 “D + M” versus “D”.

In a separate trial, diabetic mice dosed with oral glucose load showed a significant increase in blood glucose level and areas under the circle of blood glucose and time (AUC) compared with normal controls (Figure 4). SG significantly inhibited the increase of blood glucose level at 1 and 2 h. Metformin significantly inhibited this increase at 2 h after oral glucose load. The increase in AUC was significantly inhibited in diabetic mice after administration of SG or metformin. These results suggested that SG can ameliorate glucose intolerance in diabetes mellitus.

### 3.7. Intraperitoneal Insulin Tolerance Test

**Figure 5:**
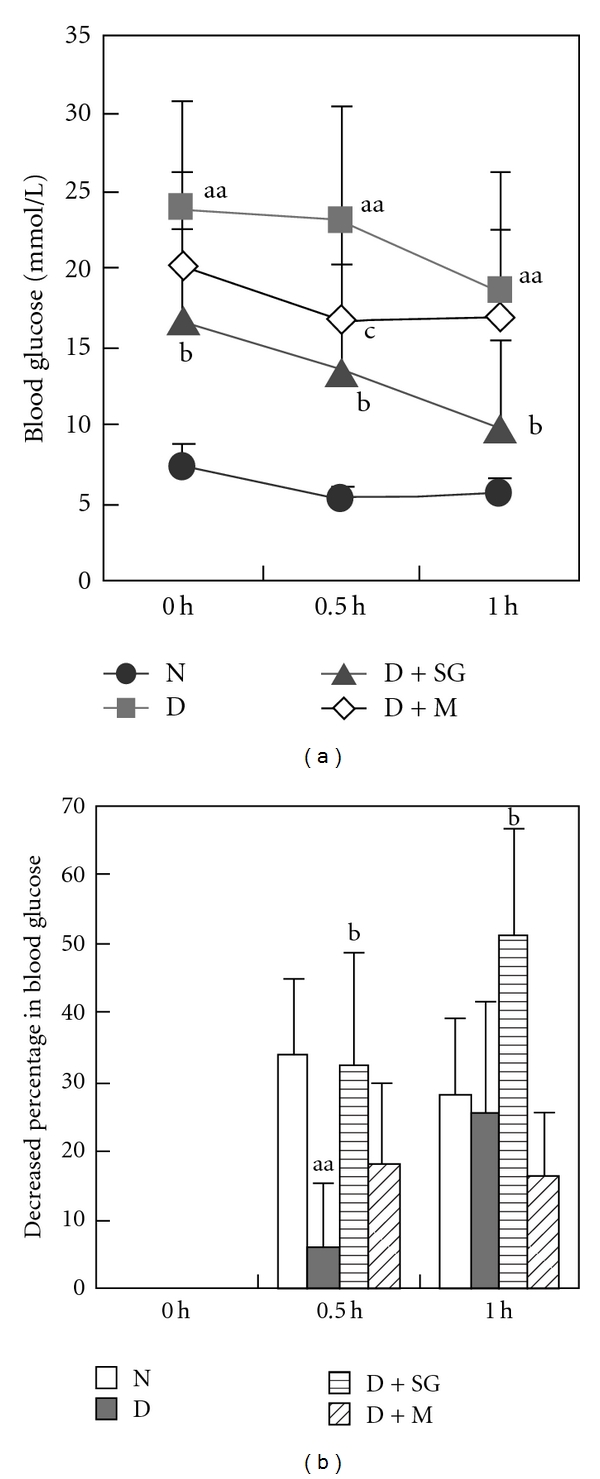
Changes in blood glucose levels with time (a) and decreased percentages of blood glucose level (b) after ip injection of insulin. N, Normal control mice; D, diabetic control mice; D + SG, diabetic mice treated with scorpion (350 mg kg^−1^ per day) combined with gypsum (350 mg kg^−1^ per day); D + M, diabetic mice treated with metformin (250 mg kg^−1^ per day). Data were expressed as mean ± SD (*n* = 6). ^aa^ *P* < .01 “D” versus “N”; ^b^ *P* < .05, “D + SG” versus “D”; ^c^ *P* < .05 “D + M” versus “D”.

In another separate trial, diabetic mice showed less decrease of blood glucose percentage compared with normal controls after ip injection of small dose of exogenous insulin (Figure 5). However, SG had significantly lowered the blood glucose level compared with diabetic controls. Metformin had no significant effect in diabetic mice. These results indicated that SG enhanced insulin sensitivity and might serve as an adjunct to insulin treatment.

### 3.8. Western Blotting

**Figure 6:**
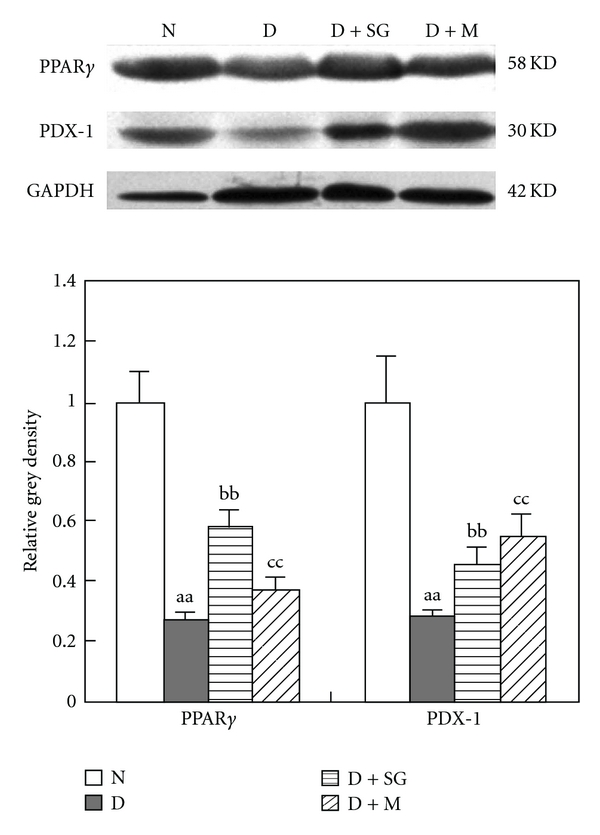
PPAR*γ* and PDX-1 expressions in pancreatic tissues of mice after 5 weeks of treatment. N, Normal control mice; D, diabetic control mice; D + SG, diabetic mice treated with scorpion (350 mg kg^−1^ per day) combined with gypsum (350 mg kg^−1^ per day); D + M, diabetic mice treated with metformin (250 mg kg^−1^ per day). Data were expressed as mean ± SD (*n* = 5). ^aa^ *P* < .01 “D” versus “N”; ^bb^ *P* < .05, “D + SG” versus “D”; ^cc^ *P* < .01 “D + M” versus “D”.

A western blotting assay was also conducted in pancreatic tissues because PPAR*γ* and PDX-1 play an important role in the development of pancreatic beta cells. In this trial, PPAR*γ* and PDX-1 expressions were significantly decreased in diabetic mice (Figure 6). However, SG or metformin significantly increased the PPAR*γ* and PDX-1 expressions. These results suggested that SG might promote the regeneration of pancreatic beta cells mediated by the mechanisms of enhancing the PPAR*γ* and PDX-1 expressions.

## 4. Discussion

Scorpion and gypsum have been utilized for thousands of years as ingredients in Chinese traditional medicines. Scorpions are mainly prescribed as an anticonvulsive, pain-relieving, anticoagulant, anticancer and immune-regulating medication in Chinese traditional medicine [20, 21]. On the other hand, gypsum is mainly prescribed as a heat-clearing, fire-purging and thirst-quenching drug [22]. Gypsum in combination with other agents has also been used to treat diabetes [23]. There have also been claims which indicated that scorpion combined with other Chinese medicines is effective in the treatment of type 2 DM patients [24]. However, these formulas consisted of more than five herbs, making the research results difficult to replicate due to poor quality control. In addition, no systematic and scientific pharmacological and mechanism assays were conducted in animal or clinical studies. To the authors' knowledge, scorpion or gypsum alone has no significant effect on hyperglycemia in diabetic animals or humans. In earlier studies, scorpion venom showed stimulation of glucagon secretion and inhibition of insulin release [25, 26]. Interestingly, in this study, we found that SG had antidiabetic effects, and further disclosed the action mechanisms which demonstrate the promotion of beta islet regeneration in STZ-induced diabetic mice. In addition to hypoglycemic activities, SG had an accompanying regulation effect on dyslipidemia in diabetic mice. SG significantly decreased total cholesterol/HDL ratio, which meant that this combination might have a beneficial effect in reducing the risk of cardiovascular diseases in diabetic individuals [27]. It also ameliorated impaired insulin sensitivity in diabetic mice. SG might also have a beneficial effect in type 2 diabetic individuals because these patients commonly display insulin resistance.

Beta cells in the Islets of Langerhans are the main organs for synthesizing and secreting insulin [28]. In this study, STZ irreversibly caused damage and loss in pancreatic beta cells. However, sulfonylureas exert a hypoglycemic effect by promoting insulin secretion in pancreatic beta cells [29]. In our preliminary trial, sulfonylureas had no effect in this animal model due to the irreversible damage in the beta islets induced by STZ (data not shown). Thus, metformin was selected as a positive control because its action mechanism was involved in a non-insulin-dependent manner. SG significantly increased both number and volume of pancreatic beta cells in pancreatic tissues, which might explain why SG increased serum and pancreatic tissue insulin levels. Furthermore, SG significantly increased the expressions of both PPAR*γ* and PDX-1. PPAR*γ* activation restores beta-cell function in diabetic mice through the reduction of endoplasmic reticulum stress and maintenance of euchromatin structure [30]. PPAR*γ* has a regulation effect on PDX-1 transcription [31]. PDX-1 governs the early embryonic development of the pancreas and the later differentiation of insulin-producing islet beta cells of the endocrine compartment [32, 33]. These results indicated that SG might exert a hypoglycemic effect by enhancing the PPAR*γ* and PDX-1 expressions and promoting regeneration or proliferation of pancreatic *β*-islets.

**Figure 7:**
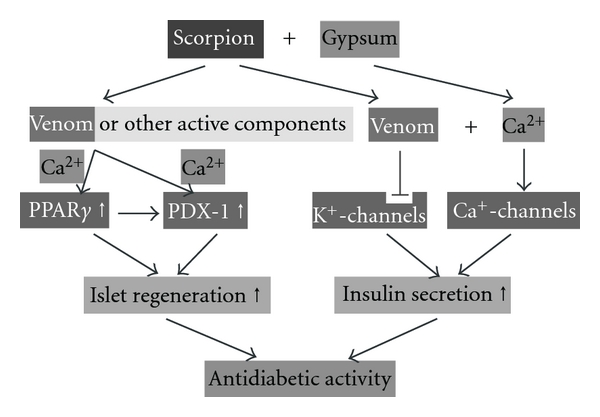
Hypothetical mechanism of action of SG in diabetic animals.

However, whether or not constituents of boiled scorpion could produce an antidiabetic effect when combined with a much diluted solution of calcium sulfate is still not known. From the observed results, a possible mechanism of action of SG is depicted in Figure 7. We believe that the tiny amount of scorpion venom which might be contained in the scorpion's body still showed potent pharmacological effect despite our removal of the toxic sting in the end of the tail containing the scorpion venom. Gypsum might reverse the untoward effects or enhance the pharmacological effect of scorpion venom. Charybdotoxin, one of the scorpion toxins, blocks calcium-activated potassium channels [34]. Blockade of calcium-activated potassium channels enhances insulin secretion, whereas blockade of Ca^2+^ channels suppresses insulin secretion [35]. Gypsum is mainly comprised of calcium (>90%). It is reasonable to suppose that the calcium in gypsum would activate Ca^2+^ channels and the charybdotoxin contained in the scorpion would inhibit K^+^ channels in pancreatic beta-islets. This dual action might have an increased effect by degrees on insulin secretion. It is also possible that Ca^2+^ in gypsum might increase the bioavailability of active principles in scorpion. The active scorpion components might be able to promote the regeneration of pancreatic beta cells. These suppositions might explain why scorpion combined with gypsum has a stronger effect than scorpion or gypsum alone. However, they should be further validated in a future study.

In addition, metformin exerted a hypoglycemic effect in diabetic patients with slight or moderate hyperglycemia, but failed in severe cases. Thus, in this study, we could not observe metformin as having significant hypoglycemic effects in the early stages of type 1 diabetic mice induced by STZ. Metformin exerts a hypoglycemic effect through the mechanism of activating AMPK [36]. In this research, we found that metformin had slight protective effect in pancreatic beta cells and that it directly promotes its regeneration in STZ-induced diabetic mice by targeting PDX-1. To date, PPAR-gamma agonists and incretin-mimetic agents seem to have this potential [5]. In this study, the effect of metformin on protecting pancreatic beta cells might be consistent with previous research [37].

## 5. Conclusion

SG has an antidiabetic effect in STZ-induced diabetic mice which might be involved in enhancing islet regeneration and promoting insulin secretion. However, the exact nature of molecular mechanisms of SG needs further investigation.

## Funding

China Postdoctoral Science Foundation (20060390460), Tertiary College Science Foundation of Nanshan, Shenzhen (2008028) and the Science Seed Foundation (2008) of the Graduate School at Shenzhen, Tsinghua University, China.

## Figures and Tables

**Table 1:** Changes in fasting serum lipid levels in mice after 5 weeks of treatment.

	Triglycerides (mmol l^−1^)		Total cholesterol (mmol l^−1^)		LDL-C (mmol l^−1^)	HDL-C (mmol l^−1^)	Total cholesterol/HDL-C
	0 week	5 weeks	0 week	5 weeks	5 weeks	5 weeks	5 weeks
N	1.05 ± 0.09	0.89 ± 0.22	4.14 ± 0.64	3.90 ± 0.57	1.73 ± 0.68	1.28 ± 0.18	3.10 ± 0.61
N + SG	1.03 ± 0.17	1.02 ± 0.27	4.10 ± 0.34	3.82 ± 0.67	1.69 ± 0.53	1.29 ± 0.19	2.97 ± 0.57
D	0.92 ± 0.12*	1.25 ± 0.21**	3.67 ± 0.41	4.72 ± 1.74	1.91 ± 0.48	0.92 ± 0.21**	5.21 ± 1.62**
D + SG	0.99 ± 0.11	0.90 ± 0.21**	3.52 ± 0.14	3.18 ± 0.51*	1.67 ± 0.21	1.03 ± 0.22	3.32 ± 0.88**
D + G	1.09 ± 0.15**	1.38 ± 0.54	3.47 ± 1.12	3.82 ± 0.74	1.91 ± 0.33	1.08 ± 0.27	3.68 ± 1.00*
D + S	1.06 ± 0.07**	1.19 ± 0.23	3.89 ± 0.37	3.89 ± 0.54	2.04 ± 0.34	1.05 ± 0.20	3.79 ± 0.71*
D + M	0.94 ± 0.09	1.54 ± 0.49	3.54 ± 0.98	3.89 ± 0.57	2.31 ± 0.40	1.08 ± 0.21	3.68 ± 0.63*

N: Normal control mice; N + SG: normal mice treated with the aqueous extracts of scorpion (350 mg kg^−1^ per day) combined with gypsum (350 mg kg^−1^ per day); D: diabetic control mice; D + SG: diabetic mice treated with scorpion (350 mg kg^−1^ per day) combined with gypsum (350 mg kg^−1^ per day); D + G: diabetic mice treated with gypsum (350 mg kg^−1^ per day); D + S: diabetic mice treated with scorpion (350 mg kg^−1^ per day); D + M: diabetic mice treated with metformin (250 mg kg^−1^ per day). Data were expressed as mean ± SD (*n* = 10). *P < .05, **P < .01 “D” versus “N”; *P < .05, **P < .01 “D + SG” versus “D”; *P < .05, **P < .01 “D + G” versus “D”; *P < .05, **P < .01 “D + S” versus “D”; *P < .05 “D + M” versus “D”.

**Table 2:** Changes in body weights, water and dietary intake in mice after 5 weeks of treatment.

	Body weight (g)		Water intake (ml g^−1^ body weight)	Diet intake (g g^−1^ body weight)
	Before treatment	After treatment
N	32.1 ± 1.3	40.7 ± 2.4	0.24 ± 0.07	0.178 ± 0.048
D	27.4 ± 4.6*	29.2 ± 3.9**	0.72 ± 0.12**	0.365 ± 0.015**
D + SG	26.9 ± 5.6	34.4 ± 2.3*	0.35 ± 0.09**	0.305 ± 0.016**
D + G	26.3 ± 2.2	30.8 ± 6.5	0.45 ± 0.08*	0.359 ± 0.020
D + S	27.1 ± 4.1	34.7 ± 3.4*	0.66 ± 0.10	0.342 ± 0.053
D + M	26.7 ± 3.3	34.9 ± 4.2*	0.40 ± 0.07**	0.345 ± 0.022

N: Normal control mice; D: diabetic control mice; D + SG: diabetic mice treated with scorpion (350 mg kg^−1^ per day) combined with gypsum (350 mg kg^−1^ per day); D + G: diabetic mice treated with gypsum (350 mg kg^−1^ per day); D + S: diabetic mice treated with scorpion (350 mg kg^−1^ per day); D + M: diabetic mice treated with metformin (250 mg kg^−1^ per day). Data were expressed as mean ± SD (*n* = 10). *P < .05, **P < .01 “D” versus “N”; *P < .05, **P < .01 “D + SG” versus “D”; *P < .05 “D + G” versus “D”; *P < .05 “D + S” versus “D”; *P < .05, **P < .01 “D + M” versus “D”.
